# Chronic Rheumatic Endocarditis

**Published:** 1969-10

**Authors:** J. F. Goodwin


### NATURAL HISTORY AND MANAGEMENT OF CHRONIC RHEUMATIC ENDOCARDITIS

**J. F. Goodwin**

### MITRAL VALVE DISEASE

In many patients with mitral valve disease of rheumatic origin there is a long latent period between the acute attack of rheumatic fever and the development of established endocarditis and symptoms. This is particularly true in pure mitral stenosis where symptoms may not begin until 20 years after the acute attack. Mitral stenosis represents a less severe degree of valve damage than does severe mitral regurgitation or mixed lesions in which the subvalvar apparatus, the chordae and papillary muscles, are involved as well as the valve cusps. In pure mitral stenosis symptoms begin gradually with dyspnoea on effort, tiredness, bronchitis in the winter, and often haemoptysis. The course tends to be one of gradually increasing disability punctuated by various crises (Goodwin, 1967).

One of the most important of these crises is pregnancy, which, because of the added haemodynamic load, converts mitral stenosis of moderate degree into a lesion of severe symptoms and serious possibilities.

Another crisis is atrial fibrillation which develops in the majority of patients with appreciable mitral valve disease by the age of 50 to 55 years and is often paroxysmal in nature initially, finally becoming established, often in the first instance with a rapid ventricular rate which leads to the development of heart failure. Embolism may occur during a paroxysm of fibrillation or at the onset of established fibrillation, and is a further serious hazard. Once atrial fibrillation has been established, patients are at risk from systemic embolism at any time. In Bannister's (1960) series systemic embolism occurred in 22 of 105 patients followed for an average of 4.5 years, while in our own series at the Royal Postgraduate Medical School over a 10 year period the incidence of probable or certain systemic embolism was 40% to 45% of 155 patients with atrial fibrillation. Sixteen patients had more than one embolism (Goodwin, 1967). While embolism is less common in free mitral regurgitation with atrial fibrillation than in mitral stenosis with atrial fibrillation, there is no difference in its incidence between mitral stenosis and mixed mitral stenotic and regurgitant lesions. The frequency of systemic embolism may be directly related to the size of the left atrial appendage (Somerville & Chambers, 1964).

Apart from causing severe incapacity or death, systemic hypertension may be related to systemic embolism as a result of multiple renal emboli, and studies by my colleagues at the Royal Postgraduate Medical School have shown a relation between atrial fibrillation, systemic hypertension, and renal infarction in patients with mitral valve disease (Obeyesekere *et al.*, 1965).

If the patient survives the hazards of pregnancy, atrial fibrillation, and embolism, the disease progresses steadily, with increasing incapacity, to the development of tricuspid regurgitation, congestive heart failure, often pulmonary embolism, and finally death, which occurs perhaps 40 years after the acute attack of rheumatic fever.

The persistent elevation of left atrial pressure due to the mitral obstruction leads to the development of interstitial pulmonary oedema and inversion of the normal pattern of pulmonary blood flow, so that the flow to the bases of the lungs becomes less than that to the apices, which is the reverse of normal. The lungs become stiff and heavy, the work of breathing increases, and this, together with the chronic venous congestion and interstitial oedema, is responsible for the dyspnoea and bronchitis. Haemoptysis is due to rupture of small varicose broncho-pulmonary anastomoses. Chronic thickening of the alveolo-capillary membranes tends to protect the patient from acute massive pulmonary oedema unless extreme surges of left atrial pressure occur, or tachycardia limits the filling time of the left ventricle and thus further elevates left atrial pressure. Thus, although in pregnancy and at the onset of acute atrial fibrillation or in the presence of a severe chest infection acute pulmonary oedema may occur, in other circumstances it is relatively uncommon in pure mitral stenosis. The elevation of pulmonary venous pressure resulting from the high left atrial pressure leads in the majority of patients to a "passive" increase in pulmonary arterial pressure, but in a minority there is active vasoconstriction in the pulmonary arterioles leading to a disproportionate rise of pulmonary arterial pressure. This "active" pulmonary hypertension may occasionally reach extreme degrees and exceptionally may exceed the systemic arterial pressure. Even in such patients, however, the changes in the pulmonary arterioles are usually limited to medial hypertrophy, and organic obliteration is rare except for some athero-sclerosis in isolated vessels and the development of atheromatous plaques in the main pulmonary artery. Thus it is well known that operations which lower the left atrial pressure are accompanied by a fall in pulmonary arterial pressure. Irreversible severe pulmonary hypertension is extremely uncommon in mitral stenosis although an incomplete valvotomy which does not succeed in reducing the left atrial pressure adequately is followed by persistent pulmonary hypertension. If the left atrial pressure can be restored to normal, as after successful mitral valve replacement, the pulmonary vascular resistance can fall to satisfactory levels (Braunwald *et al.*, 1967).

Careful regular observation can serve to detect accurately a rising pulmonary vascular resistance by observing an increase in the intensity of pulmonary valve closure, interstitial oedema, and reduction in the calibre of lower lobe vessels on radiography, and increasing right ventricular hypertrophy on the electrocardiogram.

Little has been written on the incidence of active rheumatic carditis in adult patients with established rheumatic heart disease. There can be no doubt that this occurs but detection can be extremely difficult. Wilson and Lim (1957) reported less than a 3% incidence in patients over the age of 20 years. Overt attacks are extremely rare and in our own experience we have encountered only 14 such patients over the age of 30 years, 13 having established rheumatic heart disease and clinical and serological evidence of acute rheumatic fever with carditis. We believe that subclinical active rheumatic carditis may cause the patient to deteriorate and that the true incidence is probably much higher than the figure of 2.5% in a 6 year period (Wee and Goodwin, 1966). Unexplained fever, heart failure, or other deterioration in a patient with mitral stenosis should be considered as possibly due to active carditis, although other causes such as infective endocarditis and repeated pulmonary embolism must also receive consideration.

*Diagnosis.* The diagnosis of critical mitral stenosis is not usually difficult. The first heart sound at the cardiac apex is loud and there is a loud early opening click of the mitral valve followed by a long, rumbling diastolic murmur with presystolic accentuation if the patient is in sinus rhythm. The earlier the opening snap the tighter the stenosis; and tight stenosis is usually accompanied by a full length murmur. Accentuation of pulmonary valve closure is not always a feature. Important mitral regurgitation can usually be ruled out by the absence of left ventricular enlargement and of the pansystolic murmur at the apex radiating to the axilla. In patients with pure mitral stenosis and pulmonary hypertension accompanied by tricuspid regurgitation the tricuspid murmur may be mistaken for that of mitral regurgitation. However it is usually confined to the tricuspid area or just to the left of it and does not radiate to the axilla. Pulmonary valve closure is accentuated as a result of pulmonary hypertension and there is a systolic wave in the jugular venous pulse.

### MITRAL VALVOTOMY

When a patient with pure mitral stenosis suffers from symptoms due to the disease then mitral valvotomy is indicated. In many patients however mitral stenosis may be detected clinically but symptoms may be absent, and under these circumstances operation can often be safely delayed provided that there is no evidence of interstitial pulmonary oedema, and that the patient has not suffered a systemic embolism and is still in sinus rhythm. Usually however, when the chest radiograph shows evidence of interstitial oedema there are some symptoms, or some limitation of activity. However, the risks of mitral valvotomy in skilled hands in young adults are now so small that an argument can be made for operation once the diagnosis of critical mitral stenosis has been made. This argument is strengthened in the case of young women of child-bearing age since pregnancy may precipitate symptoms or dangerous situations. With regular observation however this situation can usually be avoided.

Implicit in a decision to perform mitral valvotomy must be the presence of critical mitral stenosis; that is, a valve diameter between 0.5 cm. and 1.75 cm. Most patients who come to mitral valvotomy have a rather uniform valve size between 1 and 1.5 cm. The presence of calcification increases valve rigidity and may considerably reduce the effective valve orifice even though the diameter may not be of the smallest.

Turner and his colleagues (Kitchen *et al.*, 1968) consider that early valvotomy will prevent or delay progressive damage to the heart and pulmonary vasculature and advise early valvotomy on the diagnosis of critical stenosis.

Once atrial fibrillation has developed and the patient is at risk from embolism the indications for operation become urgent. Mitral valvotomy reduces the incidence of systemic embolism in the months and years following the operation and should always be performed under anticoagulant cover to prevent embolism at the time of, or immediately after, the operation (Smith *et al.*, 1965). If a patient who is known to have critical mitral stenosis develops atrial fibrillation, anticoagulants should be started immediately to protect from embolism, and the valvotomy should be carried out not less than 6 weeks from the start of the anticoagulant therapy. Following valvotomy the patient should be converted to sinus ryhthm by direct current countershock, which if performed in patients with recent atrial fibrillation is successful in a high proportion.

Ideally all patients with critical mitral stenosis should be placed on anticoagulants as they approach the age when atrial fibrillation is likely to develop, but unfortunately the difficulties of long term anticoagulant therapy do not at the moment justify this policy. It must be admitted that the risks of embolism at the onset of atrial fibrillation are an added reason for advising valvotomy if the valve is suitable, even in the absence of symptoms.

Age alone is no bar to successful mitral valvotomy and in our experience the mortality is similar in patients over the age of 50 years to that in younger patients, provided that selection is meticulous. The presence of any mitral regurgitation, valvar calcification, other valve lesions, chronic lung disease, degenerative arterial disease, or diabetes mellitus, increases the risks of valvotomy and diminishes the degree of improvement (Kulbertus and Kirk, 1968).

*Valvotomy in Pregnancy* is urgently indicated if, despite medical treatment, the patient is dyspnoeic on slight exertion and has persistent rales at the bases of the lungs, persistent tachycardia, or haemoptysis. In such patients pulmonary oedema may occur with alarming and sometimes catastrophic suddenness. There should be no hesitation in recommending mitral valvotomy since the operation rarely disturbs the foetus and the risks to the mother appear to be no greater than in the non-pregnant state (Boyle *et al.*, 1964). These considerations apply particularly to patients at the beginning of pregnancy when the maximum circulatory load is yet to come, but it should be remembered that even if the patient is in the last trimester and seems to be in little danger from her mitral stenosis *during pregnancy*, pulmonary œdema can develop after delivery.

### CONTRAINDICATIONS TO MITRAL VALVOTOMY

The two absolute contraindications are lack of severity of stenosis on the one hand and an unsuitable valve on the other. Extreme rigidity, heavy calcification, or considerable regurgitation all make mitral valvotomy unsatisfactory or impossible. Adverse factors limiting the effectiveness of the operation are slight degrees of rigidity and calcification, slight mitral regurgitation, and calcification in the left atrium which makes access to the valve difficult.

Pulmonary hypertension, so far from being a contraindication to mitral valvotomy, is a positive indication provided that the valve is suitable, since reduction of left atrial pressure is the only way of lowering the pulmonary arterial pressure. If the valve shows adverse features, however, consideration may have to be given to valve replacement rather than valvotomy.

### THE RESULTS OF MITRAL VALVOTOMY

The results of mitral valvotomy are extremely satisfactory, the mortality in skilled hands for many years having been less than 4%. The morbidity associated with the operation is usually trivial. When the mitral valve is calcified the operative mortality is higher and in the series of Kitchener and Turner (1967) was 17% as opposed to 4% in patients without calcification.

*Mitral Re-stenosis.* True mitral re-stenosis is not common, occurring in 4% of 100 patients investigated by Ross and Cleland (1969), and 12% of 300 patients studied by Obeyesekere and Goodwin (1967). Re-stenosis is less common after a full split by the trans-ventricular dilator. Symptoms of important re-stenosis usually begin 2-5 years after excellent surgical relief of stenosis. Symptoms occurring soon after the operation suggest significant mitral regurgitation and both lesions may coexist after valvotomy.

*Mitral Valvotomy in Children.* Tight mitral stenosis with severe pulmonary hypertension is common in Asiatic and Middle Eastern countries (Al-Bahrani *et al.*, 1966). Valvotomy is indicated on the usual grounds and is usually most effective. The incidence of re-stenosis is not fully known.

### MITRAL REGURGITATION

When the mitral valve has been severely and repeatedly damaged by rheumatic involvement, mitral regurgitation may be severe at an early stage and symptoms may develop within a relatively short interval after the acute attack of rheumatic fever, without the long latent period that is often present in mitral stenosis. Patients with severe rheumatic mitral regurgitation often have a history of repeated attacks of rheumatic fever, as opposed to patients with mitral stenosis in whom there is often a history of one attack only, or of none at all.

Many patients with mitral regurgitation, however, remain symptomless for long periods if the valve disease is not of an extreme degree. Freedom from severe dyspnoea is due to the size of the left atrium, which damps the raised pulmonary venous pressure which in full MR is mainly due to a large 'V' wave, the mean pressure being only slightly elevated. Braunwald and Awe (1963) have shown that when the left atrium is very large the pulmonary venous pressure may be low or even normal. When the left atrium in mitral regurgitation is smaller, the compliance is less, and pulmonary venous pressure may be higher, with more dyspnoea.

Symptoms commonly increase or develop with the onset of atrial fibrillation, which may develop at an earlier stage than in mitral stenosis because of the size of the left atrium and the severity of the rheumatic involvement. The tendency to atrial fibrillation tends to be inversely proportional to the size of the left atrium.

Patients with moderate mitral regurgitation are at greater risk from infective endocarditis than those with pure stenosis, and infection of the mitral valve may cause serious deterioration by further damaging the valve cusps or by causing chordal rupture and additional subvalvar mitral regurgitation.

Systemic embolism is less common in pure regurgitation than in pure stenosis, but in mixed valve lesions the incidence is equal to that in pure stenosis. Systemic embolism usually occurs with established atrial fibrillation but it is well known that emboli can occur at the onset of the arrhythmia or during paroxysmal bursts of fibrillation so that the patient may be in sinus ryhthm when seen after the embolism has occurred.

It is important to realise, in considering the natural history of mitral valve disease, that many patients with moderately severe mitral regurgitation may remain with only moderate symptoms and without deterioration for many years, unless one of the complications such as atrial fibrillation, infection, systemic embolism, or recurrent rheumatic carditis occurs.

### SURGICAL TREATMENT OF MITRAL REGURGITATION

In some patients the regurgitant mitral valve may be repaired by one or other of the techniques of annuloplasty (Wooller *et al.*, 1962) if the anterior cusp of the mitral valve remains mobile and calcification is not excessive. Buttressing of the shrivelled cusp with a pericardial patch has been suggested as a more attractive procedure, and some operations are strikingly successful although there is a tendency for the mitral regurgitation to recur (Cleland, 1967).

In most patients, however, it is necessary to replace the mitral valve, the most commonly used prosthesis being the Starr-Edwards caged ball valve. Heterograft aortic valves in a supporting frame have been used by Tonescu *et al.* (1967) and further recent experience suggests that these prostheses are promising and are likely to be attended by a much lower incidence of thrombosis and embolism than the ball valve or similar mechanical prostheses. Using an inverted aortic valve homograft in the mitral position stretched on a special metal ring inserted in the atrial side of the mitral area, Ross (1969) reported a total of 24 patients with 2 deaths.

The early mortality for mitral valve replacement is now low. Starr *et al.* (1967) reported an 18% mortality for isolated mitral valve replacement, and the figure is now in the region of 10%.

*Indications for Mitral Valve Replacement.* In view of continuing uncertainty about the long-term results of mechanical prostheses, operation should only be advised in patients with severe disease and symptoms, and a life-expectancy of 5 years or less. The presence of severe pulmonary vascular disease is an important indication, since reduction of left atrial pressure is the only effective means of reversing the progressive damage to the pulmonary vasculature. Patients with moderate symptoms and without haemodynamic deterioration or severe pulmonary vascular disease should be kept under observation, and operation should be deferred.

Severity of the mitral regurgitation, heart failure, or other valve lesions, should not constitute a contraindication for there are few patients who should be considered as too ill for surgical relief. However it must be admitted that patients with prolonged congestive heart failure, cardiac fibrosis of the liver, impaired renal function, or degenerative arterial disease, constitute a very high-risk group and it is these patients who tend to increase the mortality figures in any series.

### RHEUMATIC AORTIC VALVE DISEASE

In most patients with aortic valve disease, mitral valve disease is present also and often dominates the picture and determines the natural history. When isolated aortic valve disease occurs as a result of the rheumatic process it usually takes the form of mixed aortic stenosis and regurgitation, sometimes of free regurgitation, and only exceptionally of lone aortic stenosis. The history of aortic stenosis and regurgitation is often somewhat similar to that of mitral regurgitation in that patients tend to remain well and often more strikingly free from symptoms. Sudden deterioration is not uncommon and may take the form of attacks of left ventricular failure, dyspnoea on exertion due to left ventricular failure, or angina. These symptoms may be followed by right ventricular failure, and once this has occurred the prognosis becomes extremely bad and death may be expected within

6 months to 2 years. Operation is not usually recommended in patients without any symptoms whatever though the presence of increasing cardiac enlargement on radiological and electrocardiographic grounds may constitute an indication in some patients. Usually, however, operation is indicated at the onset of significant symptoms of dyspnoea, cardiac pain, or syncope, in patients in whom the physical signs indicate severe aortic valve disease (whether mixed or regurgitant alone). In patients with severe aortic stenosis with cardiographic signs of left ventricular hypertrophy and a pressure gradient of more than 50 mm. Hg. across the aortic valve, operation may be indicated in the absence of symptoms, particularly in younger patients in whom sudden death may occur (MacDonald, 1967).

The aortic valve must be replaced by a homograft, a heterograft or a mechanical prosthesis. The two types now favoured are the homograft on the one hand and the Starr-Valve caged ball prosthesis on the other. Reports from Starr's group (Lewis *et al.*, 1966) and from the National Heart Hospital, London, by Ross and his colleagues (Cleland *et al.*, 1969) indicate that the early and late cumulative mortality was around 24%, but in Lewis's series it later fell to 13%. Our own experience at the Royal Postgraduate Medical School revealed an early mortality of 7% in 1966 and 5% in 1967. The incidence was 15% if later deaths are included also (Cleland *et al.*, 1968).

Thus the initial results of aortic valve replacement with a mechanical prosthesis are highly encouraging; but later problems of infection, thrombosis, and embolism, account for a continued incidence of morbidity and mortality. Traumatic haemolytic anaemia has been well documented following valve replacement but more recently fatty infiltration of the silicone rubber ball leading to change in its contour known as 'ball variance' has been described. The development of haemolytic anaemia, and recurrence of cardiac symptoms, and the disappearance of the ejection click of the normal ball are important clues.

### TRICUSPID VALVE DISEASE

Tricuspid regurgitation is commonly secondary to mitral valve disease with pulmonary hypertension and atrial fibrillation, especially when the mitral valve lesion is predominantly regurgitant. Usually control of the ventricular rate and medical treatment for heart failure will produce remission of symptoms but in some patients severe tricuspid regurgitation remains and is likely to be due to organic disease. Tricuspid stenosis is usually accompanied by severe mitral and aortic valve disease, but may not produce great disability for many years. Its presence is suggested by a dominant 'A' wave in the jugular venous pulse without marked right ventricular enlargement or pulmonary hypertension, and a tricuspid diastolic murmur. Right atrial enlargement on the electrocardiogram and chest film is notable.

Moderate degrees of tricuspid valve disease do not warrant surgical treatment, but severe stenosis with a low cardiac output requires valve replacement, with ball valve or homograft. Usually the aortic and mitral valves will require replacement also. The results of triple valve replacement are similar to those of double valve replacement.

Severe intractable tricuspid regurgitation requires annuloplasty or replacement, but Braunwald *et al.* (1966) have shown that if the left atrial pressure is adequately lowered by mitral valve replacement, pulmonary hypertension will remit, and tricuspid valve function will improve markedly in many patients. Usually the decision whether the tricuspid valve requires attention must be left to the surgeon at the time of operation for mitral disease.

### References

Goodwin, J. F. (1967). Proc. Roy. Soc. Med., *60*, 1009.

Duvoisin, G. E., Wallace, R. B., Ellis, F. H. Jr., Anderson, M. W. and McGoon, D. C. (1968). Circulation, 37, *11*, 75.

Bannister, R. G. (1960). Lancet, *2*, 329.

Somerville, W. and Chambers, R. J. (1964). Brit. Med. J., *2*, 1167.

Obeysekere, I., Dulake, M., Demendash, H. and Hollister, R. (1965). Brit. Med. J., *2*, 441.

Braunwald, N. S., Ross, J. Jr., and Morrow, A. G. (1967). Circulation, Suppl. *1*, 63.

Wee, A. S. T. and Goodwin, J. F. (1966). Lancet, *2*, 239.

Wilson, M. G. and Lim, W. M. (1957). Circulation, *16*, 700.

Kitchin, A., Logan, A. and Turner, R. (1968). Abstracts of Scientific Communications Vth European Congress of Cardiology, Athens, p. 204.

Smith, B., Umathapathy, A., Bentall, H. H. and Cleland, W. P. (1965). Brit. Heart J., *2*, 1167.

Kulbertus, H. and Kirk, A. R. (1968). Brit. Med. J., *1*, 274.

Boyle, D. Mc.C., O'Donnell, M. J., Pantridge, J. F. (1964). Brit. Heart J., *26*, 337.

Kitchin, A. and Turner, R. (1967). Brit. Heart J., *29*, 137.

Braunwald, E. and Awe, W. C. (1963). Circulation, *27*, 29.

Brandt, P. W. T., Roche, A. A. G., Barratt-Boyes, B. G., Lowe, J. B. (1969). Thorax, *24*, 129.

Wooler, G. H., Nixon, P. G. F., Grimshaw, W. A., Watson, D. A. (1962). Thorax, *17*, 49.

Starr, A. and Edwards, M. L. (1961). Ann. Surg., *154*, 726.

Ionescu, M. I., Wooler, G. H., Smith, D. R., Grimshaw, V. A. (1967). Thorax, *22*, 305.

Starr, A., Herr, R. H., Ward, J. A. (1967). J. Thoracic Cardiovasc. Surg., *54*, 333.

Ross, J. K. (1969). Communication to the British Cardiac Society. To be published.

McDonald, E. L. (1967). Proc. Roy. Soc. Med., *60*, 1015.

Herr, R. H., Kloster, F. E., Sezai, Y., Starr, A. (1967). Circulation, *36*, 11, 141.

Cleland, W. P., Goodwin, J. F., McDonald, L., Ross, D. (1969). Medical and Surgical Cardiology. Blackwells, Oxford.

Al-Bahrani, I. R., Thamer, M. A., Al-Omeri, M. M., Al-Nauman, Y. D. (1966). Brit. Heart J., *28*, 824.

